# Change in Mechanical Property of Rat Brain Suffering from Chronic High Intraocular Pressure

**DOI:** 10.3390/bioengineering12080787

**Published:** 2025-07-22

**Authors:** Yukai Zeng, Kunya Zhang, Zhengyuan Ma, Xiuqing Qian

**Affiliations:** 1 School of Biomedical Engineering, Capital Medical University, Beijing 100069, China; 2 School of Basic Medical Sciences, Capital Medical University, Beijing 100069, China; 3 Beijing Key Laboratory of Intelligent Diagnosis Technology and Equipment for Optic Nerve-Related Eye Diseases, Beijing 100069, China

**Keywords:** high intraocular pressure, brain, mechanical properties, indentation test

## Abstract

Glaucoma is a trans-synaptic neurodegenerative disease, and the pathological increase in intraocular pressure (IOP) is a major risk factor of glaucoma. High IOP alters microstructure and morphologies of the brain tissue. Since mechanical properties of the brain are sensitive to the alteration of the tissue microstructure, we investigate how varying durations of chronic elevated IOP alter brain mechanical properties. A chronic high IOP rat model was induced by episcleral vein cauterization with subconjunctival injection of 5-Fluorouracil. At 2, 4 and 8 weeks after induction, indentation tests were performed on the brain slices to measure mechanical properties in the hippocampus, lateral geniculate nucleus and occipital lobe of both hemispheres. Meanwhile, the brain’s microstructure was assessed via F-actin and myelin staining. Compared to the blank control group, the Young’s modulus decreased in all three brain regions in the highIOP experimental groups. F-actin fluorescence intensity and myelin area fraction were reduced in the hippocampus, while β-amyloid levels and tau phosphorylation were elevated in the experimental groups. Our study provides insight into Alzheimer’s disease pathogenesis by demonstrating how chronic high IOP alters the brain’s mechanical properties.

## 1. Introduction

Glaucoma is a trans-synaptic neurodegenerative disease [1,2,3]. The pathological increase in intraocular pressure (IOP) is an important factor for glaucoma [4]. The visual conduction pathways were impaired due to glaucoma [5,6,7]. Glaucoma patients exhibited brain changes, including cortical thinning, gray matter atrophy, and altered functional connectivity [8,9]. Gray matter volume was reduced in the right hippocampus in high-tension glaucoma patients compared with healthy controls [9]. Moreover, elevated intraocular pressure (IOP) has been proved to change cortical thickness [10]. Changes in the microstructure of tissues will affect their mechanical properties [11]. Therefore, elevated IOP may lead to alterations in the mechanical properties of the brain. It is found that the memory performance was significantly related to shear stiffness of hippocampal subfields [12]. Animal experiments have found that stiffness of the hippocampal region in Alzheimer’s disease (AD) mice decreased [13]. Chronic high IOP impairs learning and memory in rats and induces hippocampal pathology [14]. Although numerous studies have explored the association between chronic high IOP and abnormalities in brain structure and function, direct evidence regarding changes in mechanical properties remains limited. Thus, investigating the changes in brain mechanical properties under chronic high IOP is meaningful for studying the pathogenesis of AD.

Neurodegenerative diseases involve remodeling of the brain’s microstructure, which leads to changes in the mechanical properties of the brain [11,15]. Brain tissue mechanical properties change in multiple sclerosis [16,17], AD [18,19], and demyelination [20,21]. Changes in the mechanical properties depend on disease stage [22]. The differences in the microstructure of different region of the brain lead to variations in mechanical properties [23]. Therefore, elevated IOP may lead to alterations in the mechanical properties in different regions of brain.

There have been many methods to determine the mechanical properties of the brain. Systematic mechanical testing methods were used to research the mechanical properties of the brain, such as compression, tension and shear test [24,25,26]. Tension tests on cylindrical samples of diameter ∼30 mm and height ∼10 mm for the porcine brain showed that the initial shear modulus was 842 Pa [25]. Rotational shear experiments [27] were conducted to obtain the mechanical properties of the porcine brain. In addition, combined shear, compression, and tension loadings were conducted on human brain tissues, in which a one-term Ogden model was fitted with a shear modulus of 0.4–1.4 kPa [28]. In order to obtain the mechanical properties of the brain in vivo, ultrasound elastography and magnetic resonance elastography were used to obtain the mechanical properties of the brain [29,30,31]. However, the resolution of in vivo mechanical property testing of the brain still needs to be improved [32]. Indentation tests can conveniently obtain the mechanical properties of different regions of materials and have been widely applied to measure the mechanical properties of brain tissue [33,34,35]. Therefore, we will use indentation testing to measure the mechanical properties of different brain regions after different durations of elevated IOP. 

The primary objective of the present work is to assess the mechanical properties of brain tissue variations over a continuous 8-week period of high IOP. We induced chronic high IOP in a rat model by cauterizing the episcleral venous with 5-fluorouracil (5-Fu) injection. At 2, 4, and 8 weeks after model induction, we obtained mechanical properties from indentation tests in multiple regions, including the lateral geniculate nucleus (LGN), occipital lobe (OL) and hippocampus of both hemispheres. We focus on these regions, because LGN and OL are related to the visual conduction pathway while hippocampus is related to cognitive functions, which are impaired for AD patients [36,37,38]. To assess the microstructure of the brain, we used F-actin fluorescence staining to obtain changes in the cytoskeleton of cells in different regions of the brain. We also assessed the changes in β-amyloid (Aβ) and phosphorylated tau protein (p-Tau) in the hippocampus regions, as Aβ and p-Tau are the hallmarks of AD. This study is the first to quantify changes in Young’s modulus in rat brain regions (hippocampus, LGN and OL) under chronic high IOP using indentation tests, providing new insights into the potential link between high IOP and AD.

## 2. Materials and Methods

### 2.1. Animals

All animals were obtained from the Experimental Animal Department of the Capital Medical University. They were housed libitum and maintained in an air-conditioned room in a 12 h light/12 h dark cycle, strictly following the ARVO Statement for the Use of Animals in Ophthalmic and Vision Research. The animal study protocol was approved by the Institutional Animal Care and Use Committee of the Capital Medical University.

In this experiment, a total of 30 Sprague-Dawley (SD) rats were utilized and categorized into four groups: a blank control group and three chronic high IOP experimental groups evaluated at the 2nd, 4th, and 8th week after high IOP induction. The chronic high IOP groups were designated as the 2-week (2 W), 4-week (4 W), and 8-week (8 W) groups based on post-induction duration. The right eye was designated as the experimental eye, whereas the left eye served as the contralateral control eye. The blank control group consisted of 9 samples, with no treatment applied to either eye. The experimental groups comprised 21 samples with elevated IOP for measuring mechanical properties in different brain regions (n = 6, 9, and 6 for 2 W, 4 W, and 8 W group, respectively). After the mechanical property measurements were completed on one half of the brain tissue, the other half of the brain tissue from the same anatomical location was taken for subsequent experiments. Three rats were randomly selected from each group (blank control and three experimental groups) for immunofluorescence staining to assess F-actin changes in the LGN, OL, and hippocampus regions. For myelin, Aβ, and p-Tau alterations, we focused specifically on the right hippocampus across different durations of high IOP (2, 4, and 8 weeks).

### 2.2. Rat Model with Chronic High IOP

The rat model with chronic high IOP was induced through a combination of episcleral vein cauterization and subconjunctival injection of 5-Fu (Nuobang Pharmaceutical Co., Shanghai, China). This method has been described in previous studies [39,40,41]. The rats were anesthetized by intraperitoneal injection of 1% pentobarbital sodium at a dose of 30 mg/kg. Then, three 2 mm incisions were made through the conjunctiva and Tenon’s capsule at the limbal periphery in the dorsal, ventral, and temporal quadrants of the right eye. The left eye was used as the contralateral control eye. The radial incisions were extended posteriorly to expose the episcleral and vortex veins. Next, a hand-held ophthalmic cautery device was used to cauterize three vortex veins and two major episcleral veins, which successfully elevated the intraocular pressure. To prevent neovascularization, 5-Fu (2.5%, 2.5 mg) was injected at the medial canthus. After that, topical ofloxacin eye drops were applied to prevent inflammation.

After high IOP was induced in rats, IOP was measured every three days using a TonoLab Rebound Tonometer (Icare, Vantaa, Finland). To eliminate the influence of circadian rhythms, the IOP measurements of awake rats were arranged between 10 a.m. and 12 a.m. If the IOP was below 25 mmHg, the cauterization procedure was carried out once more. Otherwise, only 5—Fu was injected.

### 2.3. Mechanical Property Measurement of Rat Brain

**Figure 1:**
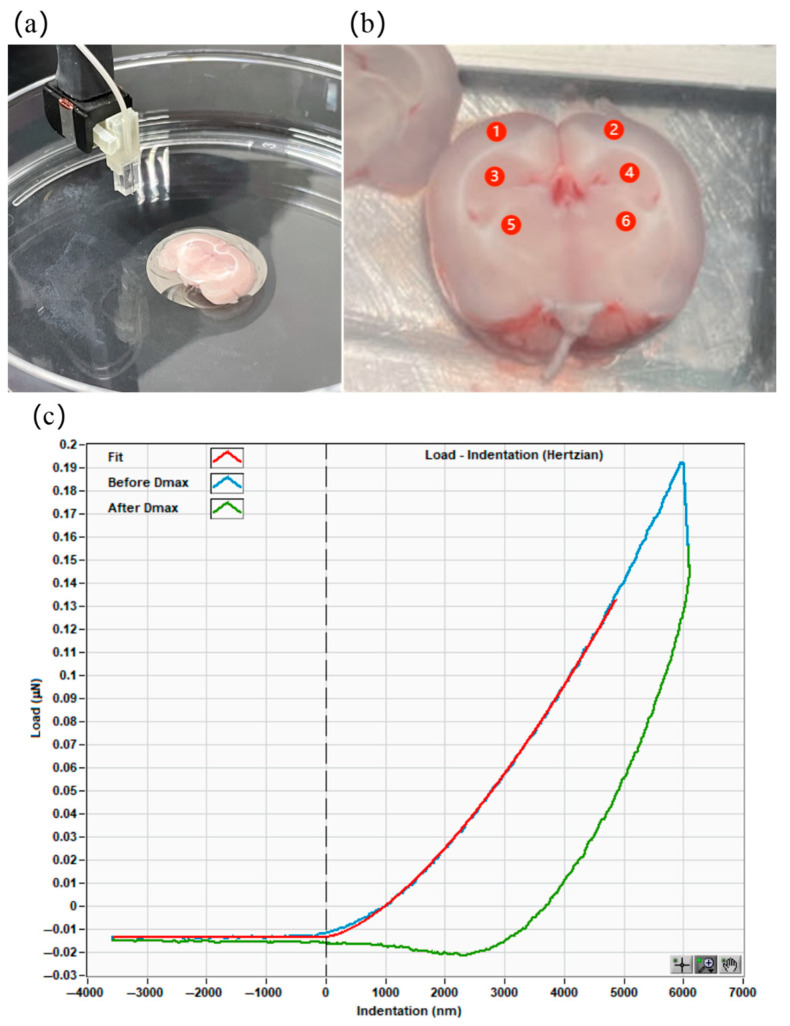
Indentation test and regions of interest. (**a**) Glue the sample slice into a Petri dish and (**b**) identify the regions of interest for indentation test, i.e., ① left OL, ② right OL, ③ left hippocampus, ④ right hippocampus, ⑤ left LGN, ⑥ right LGN. (**c**) Example of force–displacement curve in indentation test.

After 2, 4, or 8 weeks of high IOP, SD rats were euthanized by cervical dislocation. The brains were quickly dissected and soaked in ice-cold bicarbonate buffer. Coronal slices with a thickness of 1 mm at bregma −4.48mm were prepared using a Dosaka DTK-1000 N vibrating microtome (Dosaka, Kyoto, Japan). These slices were then attached to tissue-adhesive-coated dishes (BD Biosciences, Franklin Lakes, NJ, USA) filled with HEPES-buffered artificial cerebrospinal fluid (Figure 1a).

The mechanical properties in the regions of interests, such as the OL, hippocampus, and LGN (Figure 1b), were measured using a Nanoindenter (Piuma Chiaro, Optics11, Amsterdam, The Netherlands). In the test, a spherical indenter with a tip radius of 29 μm and a cantilever with a spring constant of 0.45 N/m were used. The indentation depth was set as 10 μm. Then, the force–displacement curves could be obtained (Figure 1c). The Young’s modulus could be calculated by fitting the force–displacement curves using the Hertz contact model [42]. All measurements were completed within 6 h after the rats were sacrificed.

### 2.4. Immunofluorescence Staining

Coronal brain slices were fixed in 4% paraformaldehyde, dehydrated, and embedded in OCT compound (Sakura, Finetek USA, Torrance, CA, USA). Then, they were sectioned into 12 μm thick slices. After being washed with phosphate-buffered saline (PBS) and undergoing microwave antigen retrieval in citrate buffer, the sections were incubated overnight at 4 °C with primary antibodies anti-phospho-Tau (Thr181) ( Cell Signaling Technology, Danvers, MA, USA) or anti-β-amyloid (17–24) ( Sigma-Aldrich, St. Louis, MO, USA). Subsequently, the secondary antibody, goat anti-rabbit IgG (H + L) (Invitrogen, Waltham, MA, USA), was added and incubated for 1 h in the dark. After that, DAPI counterstaining was performed. Fluorescence images were captured using a Leica TCS SP8 STED 3X stimulated emission depletion super-resolution confocal microscope (Leica Microsystems GmbH, Wetzlar, Germany).

For F-actin labeling, FITC-conjugated phalloidin (Solarbio, CA1620, Beijing, China) working solution was prepared by diluting the 20 μM stock (in methanol) with PBS containing 1% BSA to a final concentration of 100 nM. Sections were incubated with the phalloidin solution for 40 min at 37 °C in a humidified chamber protected from light to prevent fluorophore quenching. After three PBS washes, nuclei were counterstained with DAPI (1 μg/mL in PBS) for 5 min.

### 2.5. Luxol Fast Blue (LFB) Staining

In order to measure myelin content, we selected luxol fast blue staining. According to the staining protocol, we preheated the Luxol Fast Blue staining solution in a 60 °C oven for 30 min prior to use. Immerse the sections in the preheated staining solution and incubate at 60 °C for 1 h. Remove excess dye by rinsing with tap water for 30 s. After staining, allow the sections to cool to room temperature. Then, rinse them with tap water for 30 s to remove excess dye. Proceed with the differentiation process. First, submerge the sections in the differentiation solution for 15 s, followed immediately by a 10-s tap water rinse. Next, perform color differentiation in 70% ethanol for 30 s, and rinse again with tap water for 10 s. Monitor the differentiation progress under a microscope at any time. If necessary, repeat the differentiation steps until the gray and white matter are clearly demarcated. Sequentially, dehydrate the sections in 95% and 100% ethanol for approximately 10 s each. Then, clear the sections three times in xylene, with each cycle lasting 5 min. Mount the sections using either neutral balsam or Canada balsam, both of which are long-lasting mounting media suitable for microscopic analysis. Two researchers participated in the interpretation of the histological investigation results. They conducted independent analyses of the histological images in a double-blind manner, ensuring the reliability of the results.

### 2.6. Statistical Analysis

Data are expressed as the mean ± standard deviation (SD). *p*-values were obtained through independent-samples *t*-tests and one-way analysis of variance (ANOVA). Differences were considered statistically significant when * indicated *p* < 0.05, ** indicated *p* < 0.01, and *** indicated *p* < 0.001. All statistical analyses were performed using GraphPad Prism 9.0 software (GraphPad Software, Inc., San Diego, CA, USA). For the Luxol Fast Blue (LFB) staining data, due to non-normal distribution confirmed by the Shapiro–Wilk test, non-parametric statistical methods were employed. Specifically, the Kruskal–Wallis test followed by Dunn’s multiple comparisons test was used to analyze differences among multiple groups, and the Mann–Whitney U test was used for pairwise comparisons. Additionally, to explore the relationships between intraocular pressure action time, elastic modulus, Aβ, and p-Tau, a correlation analysis was performed. Pearson correlation coefficients were calculated, and the results were visualized using a 4 × 4 correlation heatmap. The heatmap was generated with color intensity representing the magnitude of the correlation coefficients and asterisks indicating statistical significance (* *p* < 0.05, ** *p* < 0.01, *** *p* < 0.001). This approach allowed for a comprehensive assessment of both group differences and inter-variable relationships in the study.

## 3. Results

### 3.1. IOP Measurement Results

**Figure 2:**
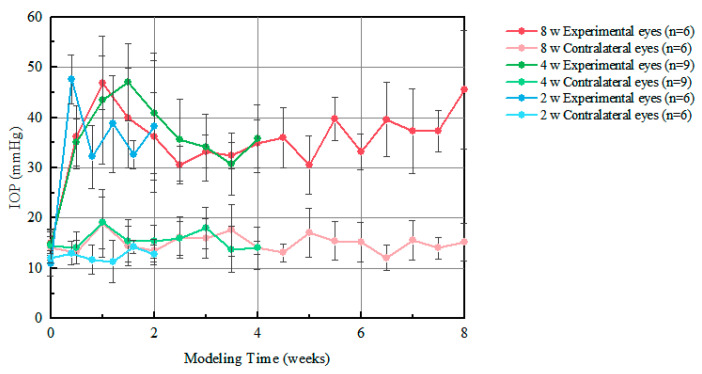
IOP measurement results.

The IOP measurement results are shown in Figure 2. IOPs of experimental groups increased significantly and sustained about 35.7 mmHg for 8 weeks, while IOPs changed little for the contralateral control eyes. 

### 3.2. Mechanical Property Measurement of Different Regions of Rat Brain

**Figure 3:**
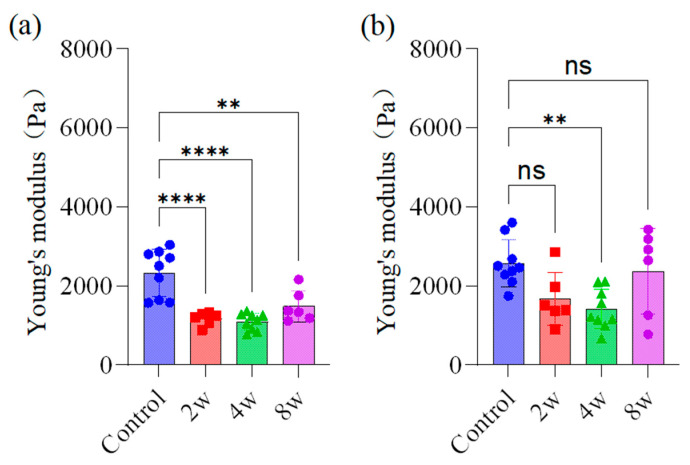

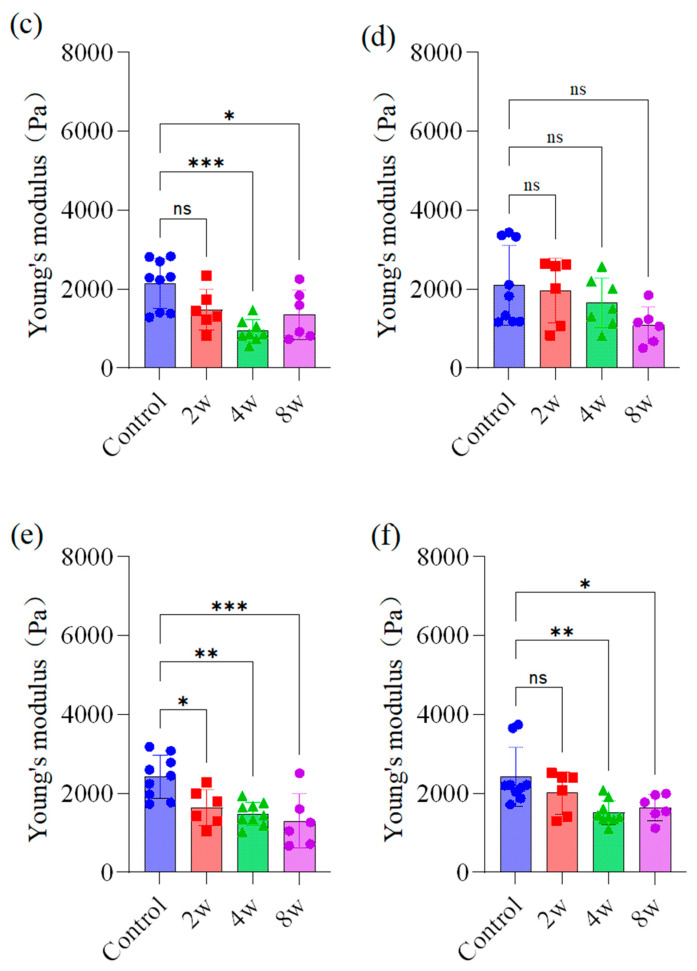
Young’s modulus in hippocampus, LGN, and OL brain regions after 2, 4, and 8 weeks of high IOP. (**a**) right hippocampus, (**b**) left hippocampus, (**c**) right LGN, (**d**) left LGN, (**e**) right OL, and (**f**) left OL. *: *p* < 0.05, **: *p* < 0.01, ***: *p* < 0.001 and ****: *p* < 0.0001 indicate statistical significance; ns indicates no statistically significant difference.

We measured mechanical properties of the rat brain when the elevated IOP of rats was sustained for 2, 4, and 8 weeks. Figure 3 shows the results in different brain regions, such as left OL, right OL, left LGN, right LGN, left hippocampus and right hippocampus. The Young’s modulus of brain tissue decreased in all three regions across different time points in high-IOP experimental groups.

The Young’s modulus of different brain regions decreased with the duration of high IOP except left LGN. From 2 weeks of chronic high IOP onward, the Young’s modulus of the right hippocampus and right OL was significantly reduced. At 4 weeks of chronic high IOP, the Young’ modulus significantly decreased in the right LGN, left hippocampus, and left OL.

After the induction of high IOP, a significant decrease in the Young’s modulus of the right hippocampal tissue was observed at 2 weeks. Under prolonged high IOP, Young’s modulus exhibited an initial decline, followed by a slight rebound to 1486.78 ± 362.47 Pa (mean ± SD) at 8 weeks (Figure 3a). The Young’s modulus of the left hippocampal tissue showed no significant difference versus the blank control group at 2 weeks of high IOP, but decreased to 1420.62 ± 477.00 Pa by 4 weeks (*p* < 0.01).

Young’s modulus of the right LGN significantly decreased after 4 weeks of high IOP (*p* < 0.001). In contrast, the left LGN showed no statistically significant differences between the high IOP experimental groups and the blank control group. In the right OL, the Young’modulus showed a progressive decrease (lowest at 8 weeks, 1302.04 ± 627.38 Pa) with different durations of high IOP. In the left OL, significant differences were detected between the blank control and high IOP experimental groups at 4 and 8 weeks, but not at 2 weeks.

### 3.3. Morphological Changes of Rat Brain with Different Durations of High IOP

**Figure 4:**
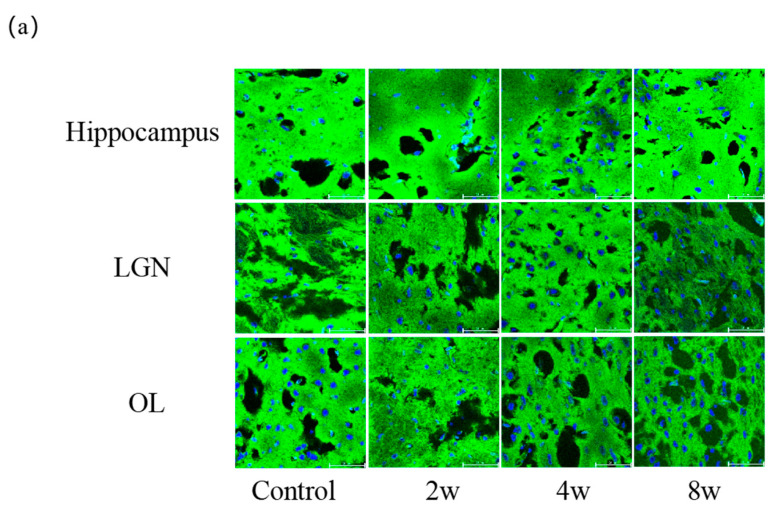

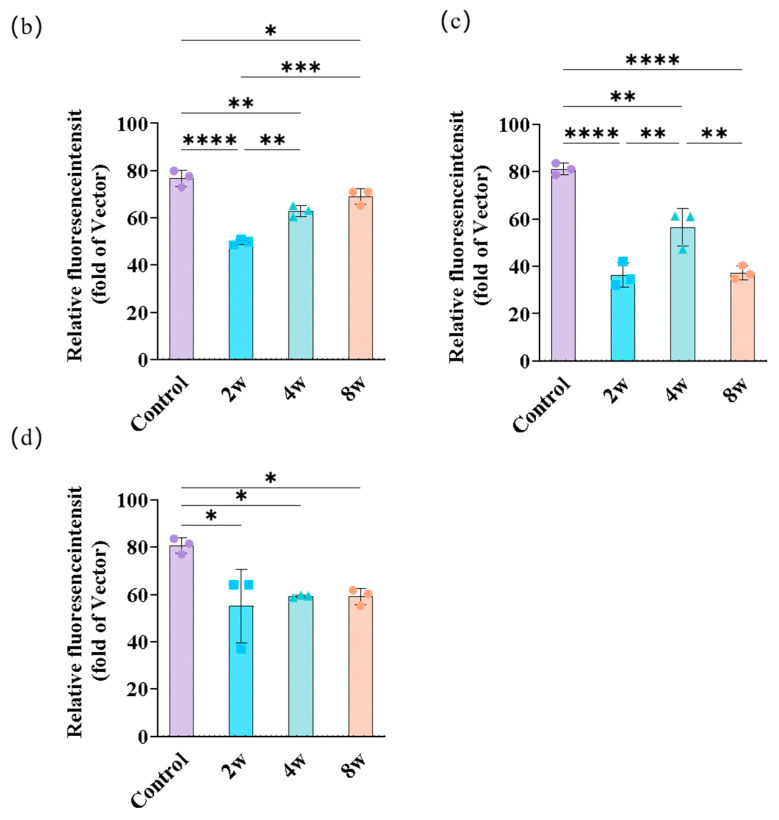
Representative confocal images of brain slices with F-actin (green) and nuclear DAPI (blue) of 2W, 4W, and 8W groups and blank control group in different regions including right LGN, right OL and right hippocampus (**a**). The relative fluorescence intensity of F-actin was assessed from different groups in (**b**) right hippocampus, (**c**) right LGN, (**d**) right OL. Scale bar: 50 μm. * *p* < 0.05, ** *p* < 0.01, *** *p* < 0.001 and **** *p* < 0.0001 indicate statistical significance.

The changes in F-actin in different regions of the rat brain at the different durations of elevated IOP are shown in Figure 4. In the right hippocampus, F-actin is distributed more densely (Figure 4a). Relative fluorescence intensity of F-actin in regions of interest was assessed using ImageJ v2.3.0 (National Institutes of Health, Bethesda, MD, USA), as shown in Figure 4b–d. Statistical analysis showed that the relative fluorescence intensity of F-actin in the same region differed significantly between the blank control group and all chronic high IOP experimental groups, indicating a significant decrease in F-actin expression in chronic high IOP conditions. The relative fluorescence intensity of F-actin in different regions showed the same trend until 4 weeks. The value in the blank control group was the highest, then decreased at 2 weeks, and increased at 4 weeks compared to that at 2 weeks. From 4 weeks to 8 weeks, the relative fluorescence intensity of F-actin declines in the right LGN. Statistical difference was also observed between two chronic high IOP experimental groups for hippocampus and LGN, which means high IOP duration will affect the expression of F-actin in the all three regions of the rat brain.

**Figure 5:**
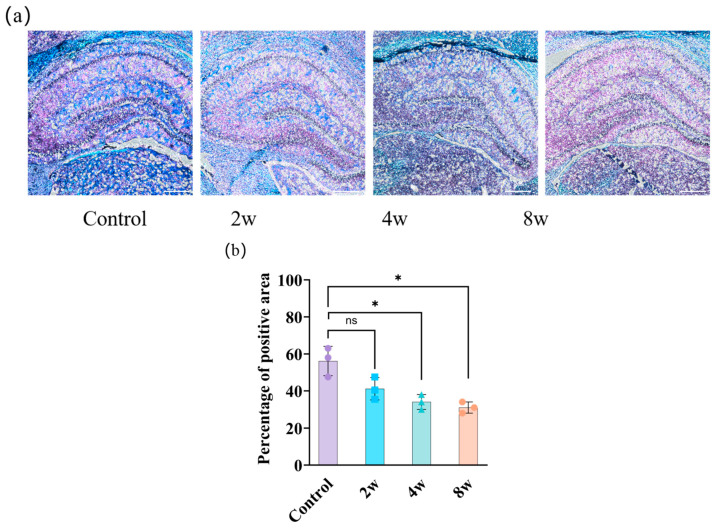
Histological images of brain slices by luxol fast blue staining for 2 W, 4 W, and 8 W groups and blank control group in right hippocampus, shown in (**a**). Myelin appears in blue, neuropil in pink, and nerve cells in purple. Area fraction changes of the myelin in right hippocampus from different groups, shown in (**b**). Scale bar: 50 μm. * *p* < 0.05 indicates statistical significance; ns indicates no statistically significant difference.

Histological characterization of the right hippocampus by luxol fast blue staining is shown in Figure 5a, where myelin appears in blue, neuropil in pink, and nerve cells in purple. In the high-IOP experimental group, myelin in the right hippocampus decreased. The area fraction values of the myelin were calculated and are shown in Figure 5b. The fraction decreased consistently from 54.67 ± 7.15% (blank control) to 39.33 ± 2.05% (2 W), 36 ± 3.27% (4 W), and 33 ± 2.45% (8 W). Significant differences were found between the blank control group and the 4W (*p* < 0.05) and 8W (*p* < 0.05) groups.

### 3.4. Aβ and p-Tau of Rat Hippocampus with Different Durations of High IOP

**Figure 6:**
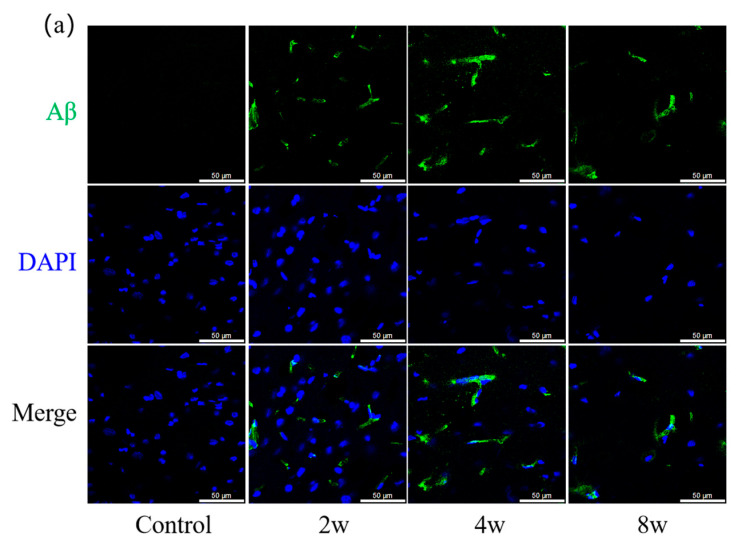

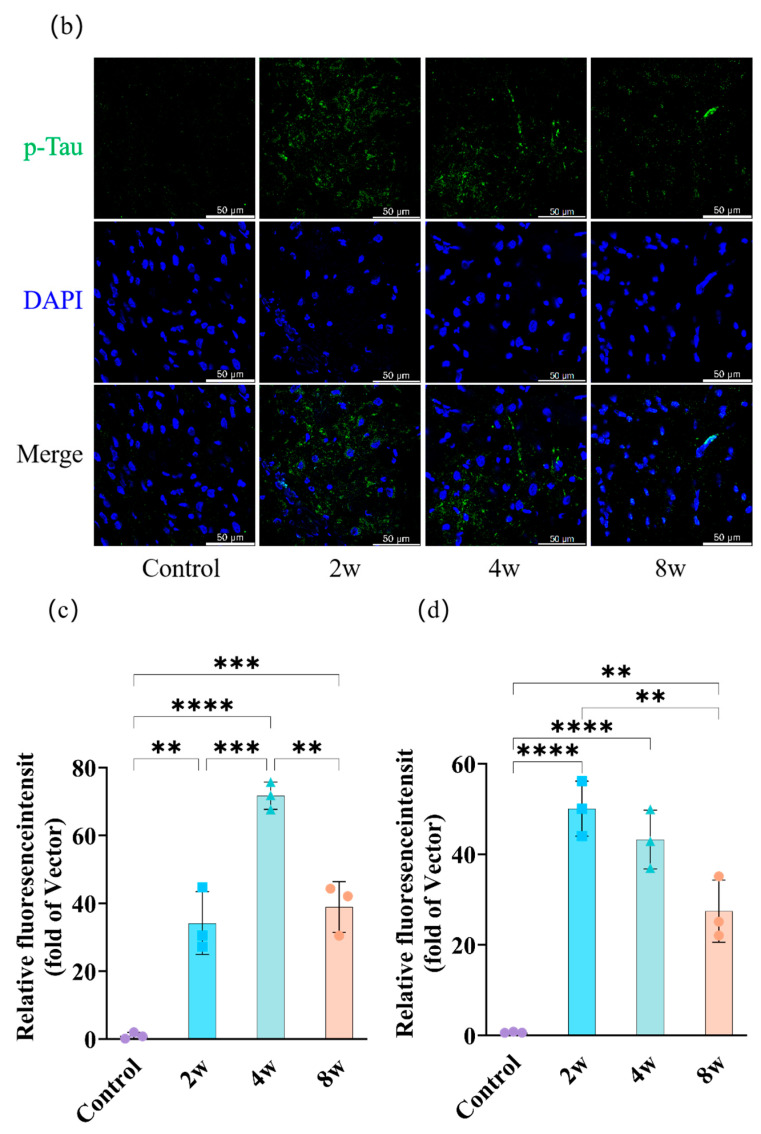
Representative confocal images of right hippocampal brain slices with Aβ (green) and nuclear DAPI (blue) staining in the blank control group and experimental groups (2 W, 4 W and 8 W) shown in (**a**). Representative confocal images of right hippocampus with p-Tau (green) and nuclear DAPI (blue) staining in the blank control group and experimental groups (2 W, 4 W and 8 W) shown in (**b**). In right hippocampus relative fluorescence intensity of Aβ (**c**) and p-Tau (**d**) was assessed. Scale bar: 50 µm. ** *p* < 0.01, *** *p* < 0.001 and **** *p* < 0.0001 indicate statistical significance.

The changes in Aβ and p-Tau in rat hippocampus at different durations of elevated IOP are shown in Figure 6. In the blank control group, neither Aβ nor p-Tau was detected, whereas in the high-IOP experimental group, Aβ accumulation was observed, as shown in Figure 6a, and p-Tau was evident, as shown in Figure 6b. Relative fluorescence intensities of Aβ were quantified and are shown in Figure 6c. High IOP induced Aβ accumulation, with intensity peaking at 4 weeks after induction and declining by 8 weeks. Statistical analysis showed that there were significant differences between the blank control group. The intensity of p-Tau was quantified and is shown in Figure 6d. The value peaked at 2 weeks post-IOP induction and subsequently declined until 8 weeks. Significant differences could be found between the blank control group and any experimental group.

## 4. Discussion

In this study, the mechanical properties and microstructural changes in multiple regions of the rat brain were evaluated following different durations of elevated IOP. Young’s modulus were deteremined by indentation tests, and F-actin was assesed by immunohistochemical staining. In addition, the area fraction of myelin, Aβ and p-Tau in the hippocampus were also assessed.

### 4.1. IOP in the Rat Model

Multiple methods have been reported for establishing rat models of chronic high IOP [43,44,45,46,47,48]. Zhong et al. reported a normal rat IOP as 17.39 ± 4.10 mmHg, based on pooled data from multiple studies [44]. Currently, no consensus exists on the required IOP elevation threshold for rat models of chronic high IOP. A model is considered valid when IOP exceeds 25 mmHg [47,48]. Thus, we maintained IOP higher than 25 mmHg to ensure model validity. A mean IOP of 35.7 mmHg sustained for 8 weeks met these criteria.

It is found that the experimental eyes showed very elevated IOP in 2 W samples in the first reading, where IOP in the 4 W and 8 W samples rose gradually to maximize at 1 week and 1.5 weeks, respectively, then gradually started decreasing. The IOP in 8 W samples again showed an elevation trend after 4.5 weeks. The variation in IOP is associated with the number of 5-Fu injections. Mean IOP values for the 2 W, 4 W, and 8 W groups were 37.7 ± 5.1 mmHg, 35.2 ± 5.1 mmHg, and 36.8 ± 4.6 mmHg, respectively. Because these values did not differ significantly across time points, this study focused on the influence of high IOP duration rather than IOP magnitude on brain mechanical properties. The potential contribution of absolute IOP levels remains to be addressed in future work. 

### 4.2. Brain Young’s Modulus Variation in Different Regions

The study found that the Young’s modulus of different brain regions in normal animals ranged from 1.1 kPa to 3.1 kPa. Previous studies using brain elastography have demonstrated that the shear elastic modulus of brain tissue decreases with age [49]. For grey matter, the shear modulus ranged approximately from 2320 Pa to 3000 Pa, and for white matter, the shear modulus ranges approximately from 2950 Pa to 3500 Pa [49]. Our results are basically consistent with the existing literature in terms of order of magnitude. For the corona radiata (coronal plane), thalamus (sagittal plane), and the corpus callosum (coronal and sagittal planes), the shear modulus values of the rat brain were between 2 and 6 KPa using a custom-built micro-indentation apparatus [50]. At an indentation depth of 3 μm using an atomic force microscope, apparent elastic modulus was several hundred Pa in the hippocampus [51]. White matter, with an average modulus of 1.895 kPa, was on average 39% stiffer than gray matter, with an average modulus of 1.389 kPa, and displayed larger regional variations using flat-punch indentation [35]. 

### 4.3. Chronic IOP Changes Brain Young’s Modulus of the Rats

We induced the chronic high IOP animal model by photocoagulation and injection of 5-FU; the glaucomatous characteristic changes in the optic nerve and retina for the model rats have been found [39,40,41]. It has been proved that glaucoma impaired the visual pathway in the brain. The number of neurons was reduced in the LGN [52,53], the LGN underwent atrophy [54], and visual cortex thickness was reduced [55] by magnetic resonance techniques. In the rat model of glaucoma, the response in the visual cortex decreased [56]. In Figure 2, brain Young’s modulus decreased with the duration of chronic high IOP in multiple regions for the model rats. As the changes in microstructure could be reflected by brain Young’s modulus, our results illustrated that the sustained high IOP impaired parts of the visual conduction pathway, such as LGN and OL. Besides the visual conduction pathways, the hippocampus was altered when subjected to high IOP in our study. Yuan [14] assessed the changes in the hippocampus of the high IOP rat model and reported learning and memory impairments along with altered microstructure.

### 4.4. Young’s Modulus Changes of the Brain with the Changes in F-actin and Myelin

The study found that the mechanical properties of different brain regions varied with the duration of elevated IOP. Changes in mechanical properties may be related to alterations in the microstructure of brain tissue [11]. Therefore, this study investigated the changes in F-actin and myelin in the hippocampal region of brain tissue under sustained high intraocular pressure. 

Our results revealed that at the cytoskeletal level, the changes in the F-actin cytoskeleton are highly correlated with changes in tissue stiffness. The fluorescence intensity of F-actin in the right hippocampus decreased by 37.5% at 2 weeks and partially recovered to 82% of the blank control group level by 8 weeks. This nonlinear change is correlated with the “decrease-rebound” trajectory of the elastic modulus. Some scholars have proposed that the cytoskeleton dominates stiffness, and actin filaments form a three-dimensional network through cross-linking proteins such as α-actinin, which serves as the fundamental mechanical unit for maintaining tissue stiffness [57,58,59]. 

The IOP means of the 4W and 8W experimental groups are similar. We believed that the changes in the mechanical properties and F-actin in the brain were the accumulated effects of chronic IOP elevation. Although the mean elastic modulus of the right LGN in the 8-week group was higher than that in the 4-week group, statistical analysis revealed no significant difference between them. However, there was significant difference between the relative fluorescence intensity of F-actin in the 8-week group and that in the 4-week group in the right LGN. The results suggested microstructural changes in the LGN preceded its biomechanical alterations and the underlying mechanisms require further investigation.

Myelin degradation is associated with changes in the mechanical properties of brain white matter [21]. Our results showed a decrease in the elastic modulus of the right hippocampus and a reduction in area fraction reduction of the myelin. Myelin is a lipid membrane that surrounds nerve fibers, primarily functioning to accelerate the conduction of nerve signals. The thickness of myelin directly affects the mechanical properties of brain tissue [21,34]. For example, in a mouse model using cuprizone-induced demyelination, it was found that the average stiffness of the corpus callosum in untreated mice was 1.1 ± 0.3 kPa. By week 3, after cuprizone treatment, the stiffness decreased to 0.6 ± 0.1 kPa, then briefly rebounded to 0.9 ± 0.3 kPa by week 6, and finally stabilized at 0.7 ± 0.1 kPa by week 9. These changes are closely related to the process of myelin removal and regeneration, indicating that variations in myelin thickness can lead to significant fluctuations in brain tissue stiffness [21]. When myelin is damaged or degenerates, the mechanical properties of brain tissue change accordingly. For example, in acute demyelinating lesions, the loss of myelin leads to a decrease in brain tissue stiffness, while during the remyelination process, tissue stiffness may gradually recover [34].

### 4.5. Chronic High IOP Induces Aβ Accumulation and Tau Phosphorylation in Hippocampus Region

AD is characterized by the presence of extracellular Aβ plaques and neurofibrillary tangles with p-Tau in the brain. Aβ and p-Tau are regarded as the hallmark pathologies of AD [60]. The Aβ deposition and tauopathy were illustrated in the LGN for a monkey model of glaucoma [61]; the effects of chronic high IOP on Aβ accumulation and tau phosphorylation were determined in the hippocampus of the model rats [14]. Our study has demonstrated the presence of AD-like pathology of Aβ accumulation and tau phosphorylation in the brain caused by chronic high IOP. These data collectively establish the existence of hallmark AD-like pathologies in glaucoma. 

**Figure 7:**
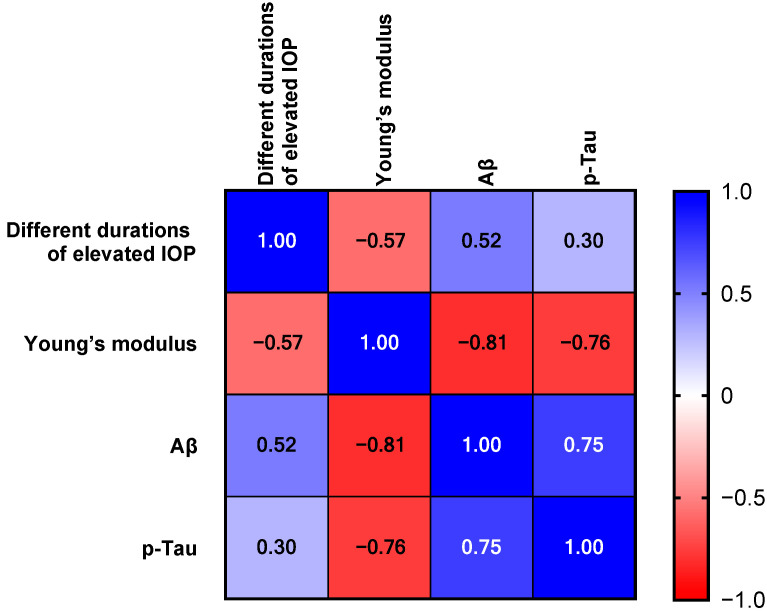
Correlation heat map illustrating the pairwise relationships among different durations of elevated IOP, Young’s modulus, Aβ, and p-Tau. The color intensity represents the magnitude of Pearson correlation coefficients, with blue indicating positive correlations and red indicating negative correlations.

We further examined the relationship between chronic high IOP and the accumulation of Aβ and p-tau (Figure 7). The 4 × 4 correlation coefficient heat map clearly reveals the strength and direction of the associations among variables. There is a strong negative correlation between elastic modulus and Aβ (r = −0.81), and a significantly negative correlation with p-Tau (r = −0.76). This suggests that as the tissue elasticity decreases, the pathological deposition of Aβ and p-Tau may increase synchronously. The intraocular pressure action time shows a moderately positive correlation with Aβ (r = 0.52), implying that long-term intraocular pressure exposure may promote the accumulation of Aβ. Aβ and p-Tau are significantly positively correlated (r = 0.75), which is consistent with the classical mechanism of their synergistic aggregation in the pathological process of Alzheimer’s disease. Overall, there are non-random associations between mechanical properties (elastic modulus, intraocular pressure action time) and Aβ, p-Tau. Negative mechanical disturbances, such as reduced elasticity and prolonged intraocular pressure, may exacerbate the deposition of pathological proteins.

The production and clearance of Aβ and p-Tau is a dynamic process. In the early stage (approximately corresponding to the 2-week time point in this paper), the early accumulation of Aβ may be associated with high intraocular pressure-induced oxidative stress activating β-secretase (BACE1) and the γ-secretase complex, thereby promoting the abnormal cleavage of amyloid precursor protein (APP) into Aβ [62,63]. The decline in the dynamic fluctuation of Aβ after 4 weeks in this paper may involve enhanced TREM2-dependent phagocytosis by microglia and Aβ degradation mediated by the repair of lysosomal acidification [64,65]. The early surge in p-Tau may be driven by Aβ-activated GSK-3β and CDK5/p25 kinases, which induce excessive phosphorylation of tau protein at the Ser396/Ser404 sites [66,67]. The subsequent decline in p-Tau is related to the recovery of protein phosphatase PP2A activity and the conversion of partially phosphorylated tau into insoluble neurofibrillary tangles (NFTs) [68,69].

### 4.6. Limitations

There are some limitations in the present work. Firstly, in our study, only LGN and OL in the visual pathway of the brain and hippocampus were measured; multiple regions were altered due to chronic high IOP and glaucoma in previous studies [8,9]. In our future work, the mechanical property changes in multiple regions of the brain will be considered. Secondly, we measured the Young’s modulus changes in the brain due to the chronic high IOP; the brain is assumed to be a linear elastic material. In future work, we will assess changes in nonlinear mechanical properties of the brain with different durations of elevated IOP. As the brain tissue is biphasic, the hydraulic conductivity of the solid phase is required [70]. We will simulate the brain with a poroelastic mechanical model with different durations of elevated IOP to better understand the flow of cerebrospinal fluid through the brain parenchyma. Finally, the resolution of brain morphological measurements is still not high enough. In the future, we plan to use scanning electron microscopy to conduct more detailed observations of the changes in brain microstructure under different durations of high intraocular pressure. 

## 5. Conclusions

In our study, we induced a chronic high IOP rat model by episcleral vein cauterization and injection of 5-FU. High IOP was sustained at over 30 mmHg for 8 weeks. Following different durations of elevated IOP, Young’s modulus of the rat brain was measured by the indentation test in regions including the LGN, OL and hippocampus. Our results indicated that Young’s modulus of the brain decreased, F-actin expression in the brain regions was altered, and the myelin area fraction in the right hippocampus was reduced. In addition, Aβ and p-Tau deposition were observed in the right hippocampus after 4 weeks of elevated IOP, indicating characteristic AD-like pathological features associated with chronic IOP elevation.

In conclusion, our study demonstrates that chronic high intraocular pressure may induce changes in the mechanical properties of brain tissue, which not only reinforces the potential pathological link between glaucoma and Alzheimer’s disease but also provides implications for the early screening of Alzheimer’s disease via eye-related examinations. Furthermore, the application of magnetic resonance elastography and other detection methods could be promoted to strengthen the prevention and monitoring of brain tissue lesions in glaucoma patients, offering a novel perspective for clinical practice in neurodegenerative disease prevention and glaucoma management.

## Data Availability

Data are contained within the article.

## Abbreviations

The following abbreviations are used in this manuscript:

IOP	intraocular pressure
5-Fu	5-Fluorouracil
LGN	lateral geniculate nucleus
OL	occipital lobe
Aβ	β-amyloid
p-Tau	phosphorylated tau protein
AD	Alzheimer’s disease
AFM	atomic force microscope
DAPI	4′,6-Diamidino-2-phenylindole
LFB	Luxol Fast Blue
OCT	Optimal Cutting Temperature
PBS	phosphate-buffered saline
